# The impact of global budget on the diffusion of innovations: the example of positron emission tomography in Taiwan

**DOI:** 10.1186/s12913-018-3731-4

**Published:** 2018-11-29

**Authors:** Che-Ming Yang

**Affiliations:** Shuang Ho Hospital, Taipei Medical University; School of Health Care Administration, College of Management, Taipei Medical University, 10F. No.172-1 Sec. 2 Keelung Rd,, Taipei, 106 Taiwan

**Keywords:** Global budget, Diffusion of innovations, Positron emission tomography (PET)

## Abstract

**Background:**

The essence of global budget is to set a cap on the total national health insurance expenditure for a year, which is one form of prospective payment systems. It has always been argued that prospective payment, such as global budgeting, will deter the development of high-tech services in the healthcare industry. The objectives of this study are to explore the impact of global budgeting on the diffusion of high tech equipment in terms of utilization by using Positron Emission Tomography (PET) as an example.

**Methods:**

The study population is the hospitals in Taiwan. We tried to compare the diffusion patterns of Computed Tomography (CT), Magnetic Resonance Imaging (MRI) and PET scanners among these hospitals by analyzing the National Health Insurance (NHI) Database from 1997 to 2010.

**Results:**

From 2004 to 2010, 79,380 PET scans in total were performed under the NHI scheme. By the year 2010, the annual reimbursed scans have reached 19,700. The volume curve of cumulative PET services resembles an S diffusion curve with the R^2^ at 0.95. The results indicated the growth of cumulative PET service volume does correspond with the innovation diffusion model. The cumulative utilizations of CT, MRI and PET demonstrate good correlation with no significant difference in their growth rates.

**Conclusions:**

Therefore, we can infer that even though PET was reimbursed after the implementation of global budgeting, its diffusion was not deterred by this cost containment measure when compared with CT and MRI in the same time span after the inauguration of the NHI.

## Background

The advance in technology improves health care outcome and elevate health care quality without a doubt. The same advance is also likely to drive up health care costs. Researches in the U.S. have long established that the changes in health care technology are responsible for half of the increase in health care costs [1]. However, managed care has been demonstrated in the literature to have the effect of deterring health care providers’ adoption of new technology [2]. The same phenomenon happens in Taiwan as well.

Taiwan has started its National Health Insurance (NHI) since 1995, which has the hall marks of universal coverage and single payer. The benefit package is quite comprehensive and the health care system has been touted as one of the best around the world. However, the NHI also has to grapple with the issue of financial sustainability. In the face of rising financial deficit, the NHI started phasing in global budgeting in the place of fee-for-service to contain cost since 2000.

The essence of global budget is to set a cap on the total national health insurance expenditure for a year. It is also one form of prospective payment systems. One of the major concerns for implementing a global budget for health care reimbursement is that it will deter the adoption and diffusion of new technology [3]. Under the restraint of a global budget, hospitals have no incentive to invest in new equipment and new technology. The purchase of new inventions more often than not means increasing operation costs for the health care industry. Under global budgeting, the health care industry is reimbursed the fixed amount stipulated one year ago depending on the premium collected. Individual health care provider will get its share according to its service volume. For a public health insurance, the premium rates remain stagnant for many years due to public resistance to premium hikes. Under such environment, there is little incentive for health care providers to increase their operation cost by adopting new technology.

Everett Rogers proposed the theory of diffusion of innovations in 1995 [4]. This theory seeks to explain how and at what rate new ideas and technology spread. Rogers proposed that diffusion is the process by which an innovation is communicated over time among the members of a social system. The characteristics of an innovation, as perceived by the members of a social system, determine its rate of adoption.

Rogers’ theory is based on the Bass diffusion model, which was developed by Frank Bass and consists of a simple differential equation that describes the process of how new products get adopted in a population [5]. An S curve model is fit to predict the uncertainty faced by new products. In recent years, Bass’ model has also been applied to study of the innovations in the Internet, mobile services etc.

**Fig. 1:**
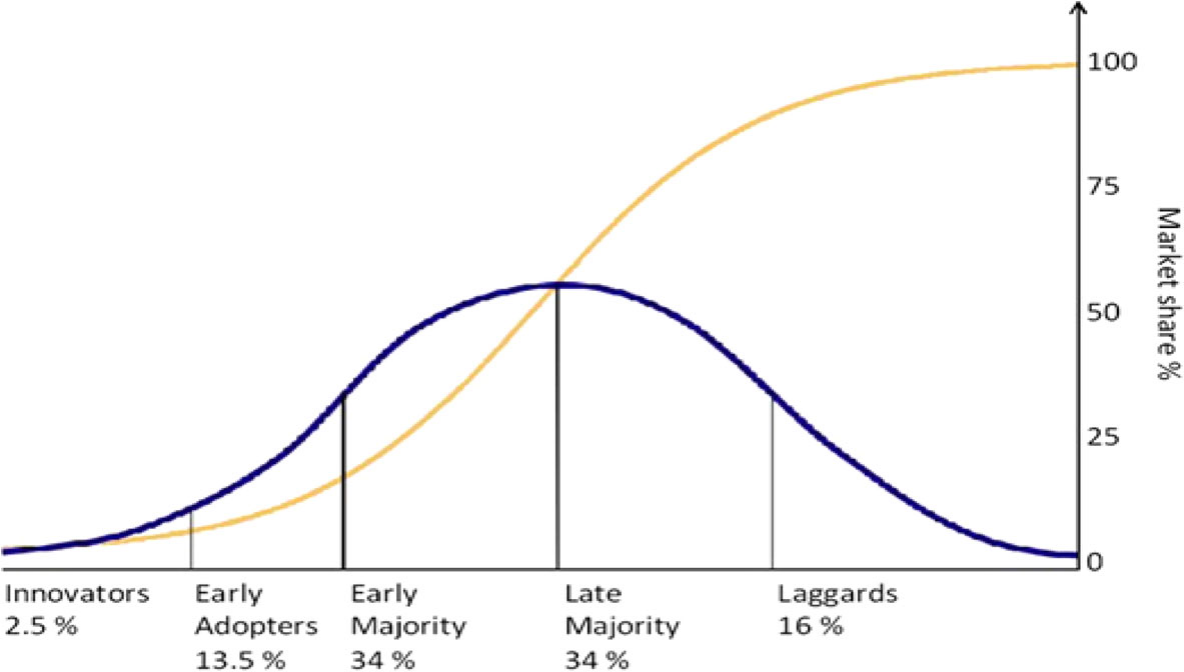
Rogers’ diffusion of innovations theory

For a successful innovation, the cumulative adopter distributions follow a bell-shaped curve over time and approach normality. The bell-shaped curve is the derivative of the S-shaped diffusion curve. The rate of adoption is defined as the relative speed at which the members of a social system adopt an innovation. Rogers divided this bell-shaped curve to characterize five categories of system member innovativeness. The five categories are: innovators, early adopters, early majority, late majority and laggards (Fig. 1).

Rogers’ theory has been widely applied in the fields of medicine and public health since 1950s. The principal researches have to do with the adoption of new drugs and new medical concepts, etc. One classical example of this kind of researches is regarding the adoption of tetracycline. Coleman et al. found that doctors who were linked in more interpersonal networks adopted the innovation more rapidly than did more isolated doctors [6].

In the healthcare field, Computed Tomography (CT) scanners, Magnetic Resonance Imaging (MRI) scanners and Positron Emission Tomography (PET) scanners are no doubt expensive high tech medical equipment. In Taiwan, CT scanner and MRI have been reimbursed by the National Health Insurance (NHI) ever since the inception of it in 1995. PET scanner is a relatively new comer. It was not reimbursed until 2004. According to the statistics of Taiwan’s Ministry of Health and Welfare, by the end of 2010, there were 329 CT scanners, 164 MRIs and 40 PETs. The changes were tabluated in Table 1 [7].

**Table 1:** The numbers of CT, MRI and PET scanners in Taiwan from 1995 to 2010

	CT		MRI		PET	
Year	Number	Increase rate year on year (%)	Number	Increase rate year on year (%)	Number	Increase rate year on year (%)
1995	236	–	39	–	–	–
1996	242	2.54%	40	2.56%	–	–
1997	272	12.40%	55	37.50%	–	–
1998	291	6.99%	56	1.82%	–	–
1999	287	−1.37%	63	12.50%	–	–
2000	308	7.32%	72	14.29%	–	–
2001	310	0.65%	81	12.5%	–	–
2002	317	2.26%	86	6.17%	–	–
2003	325	2.52%	92	6.98%	–	–
2004	318	−2.15%	102	10.87%	–	–
2005	321	0.94%	115	12.75%	22	–
2006	320	−0.31%	126	9.57%	27	22.73%
2007	318	−0.63%	130	3.17%	29	7.41%
2008	321	0.94%	142	9.23%	34	17.24%
2009	331	3.12%	156	9.86%	38	11.76%
2010	329	−0.60%	164	5.13%	40	5.26%

A CT scan makes use of computer-processed combinations of many X-ray images taken from different angles to produce cross-sectional images of specific areas. As X-ray CT is the most common form of CT in medicine and various other contexts, the term CT alone is often used to refer to X-ray CT. Whereas MRI is a medical imaging scanner used to image the anatomy and the physiological processes of the body by using strong magnetic fields to form images of the body. PET scan uses a radioactive substance called a tracer to look for disease in the body. A PET scan shows the functions of organs and tissues. The most recent trend is machines that combine the PET and CT images or the PET and MRI images.

Relevant researches mostly have to do with CT and MRI. Baker pointed out that MRI in the United States had a linear diffusion curve from 1983 to 1993 [8]. In Iran, the diffusion curve of MRI in 1991–1995 is an S curve [9]. In Korea, the diffusion curve of MRI accelerated in 1999 and maintained a linear relationship until 2004 [10]. The factors that have significant positive influence on MRI diffusion in Korea include the numbers of physician in a region, the percentage of population older than 65 years old, the number of private hospitals, the number of beds and the average income of locals; but the number of MRI in the region has a negative influence [10]. In the US, the reimbursement methods and the average per capita health expenditure are significantly related to the diffusion of MRI [11], and the differences in policy, demographic and region will affect the diffusion of CT scan [11–13].

The researches in the literature did not address the diffusion pattern of PETs and the impact of global budgeting on the diffusion of high tech medical equipment. Therefore, the objective of this study is to investigate whether global budgeting affects the diffusion patterns of high tech medical equipment such as CT scanner, MRI and PET scanner in Taiwan in light of Rogers’ theory by taking into account the effect of other influencing factors as described in the literature.

## Methods

The study subjects are the hospitals in Taiwan. We tried to compare the diffusion patterns of the three pieces of high tech equipment. The data sets that we utilized in the study are the National Health Insurance Database from 1997 to 2010, specifically including the files HOSB, CD, OO, DD and DO.

The unit of analysis is the country as a whole. The dependent variable will be the cumulative service volume of individual equipment year by year since being reimbursed by the NHI. The independent variable is the number of years after being reimbursed by the NHI in the exploratory analyses. Descriptive analyses were conducted according to the demographics of the patients who receive the particular services and characteristics of the hospitals that provide the particular service.

We used SPSS 17.0 for our statistical analyses. S statistic was applied to examine whether the diffusion curves fit an S curve model. We also applied the Chow-test to compare the diffusion curves of the three types of equipment. The Chow test is a statistical and econometric test of whether the coefficients in two linear regressions on different data sets are equal. The Chow test is often used to determine whether the independent variables have different impacts on different subgroups of the population. The assumptions of this test are homogeneity of variance and the subgroups are independently distributed.

## Results

For CT scan and MRI, we can derive our analyses from 1997 to 2010 subject to the limitation of data availability, whereas for PET from 2004 to 2010 because it was not reimbursed until 2004. As indicated before, the NHI was inaugurated in 1995 and global budgeting was implemented in 2000.

From 2004 to 2010, 79,380 PET scans in total were performed under the NHI scheme. The year by year volumes are demonstrated in Table 2. By the year 2010, the annual reimbursed scans have reached 19,700.

**Table 2:** The annual volume of CT, MRI and PET services reimbursed by the NHI as compared with the total volume revealed in the MOHW’s annual survey

Year	CT reimbursed by the NHI	MRI reimbursed by the NHI	PET reimbursed by the NHI	PET in MOHW’s annual survey	Percentage of the total PET volumes accounted by the NHI
1997	526,980	100,660	1340	N/A	
1998	601,580	126,760			
1999	695,360	166,160			
2000	653,560	160,300			
2001	665,640	199,120			
2002	779,700	240,000			
2003	828,280	271,080			
2004	969,440	317,020			
2005	948,160	313,140	5080	14,673	34.62%
2006	981,760	328,280	9780	19,242	50.83%
2007	1,086,040	366,520	11,680	26,106	44.74%
2008	1,257,160	410,700	16,000	28,334	56.47%
2009	1,295,120	481,300	15,800	30,249	52.23%
2010	1,424,820	503,880	19,700	30,632	64.31%

The MOHW has also connected annual surveys of PET services provided by hospitals since 2005. The volumes as listed in Table 2 revealed by the surveys include both the PET services reimbursed by the NHI and those paid out of patients’ own pockets. In total, 14,673 PET services were provided in 2005 nationwide, and 30,632 was done in 2010. It has doubled in 5 years and sort of plateaued in 2009 and 2010. According to the population statistics, the total population in Taiwan at the end of 2010 was 23,123,866. Therefore, the service intensity amounts to 1.32 exams per thousand persons. By comparing with the MOHW’s annual survey, we can see the NHI reimbursed percentage has risen from the 34.62% of 2005 to the 64.31% of 2010.

As indicated in Table 3, of the total 79,380 reimbursed by the NHI in our study periods, 66.14% are performed for outpatients and 33.86% for inpatients. More males have received PET scans than the female. The majority of the services are referred by the departments of internal medicine and the departments of surgery. Only 25.47% was prescribed by public hospitals, which means that the majority of PET services were performed in non-public sectors.

**Table 3:** Description of PET services reimbursed by the NHI from 2004 to 2010

Category	Volume	%
Admission		
Outpatient	52,500	66.14%
Inpatient	26,880	33.86%
Gender		
Male	44,020	55.45%
Female	35,360	44.55%
Age (years old)		
≦18	1100	1.39%
> 18 and < 65	50,360	63.44%
≧65	27,920	35.17%
Specialty		
Internal medicine	31,240	39.36%
Surgery	27,960	35.22%
Obstetrics & gynecology	1120	1.41%
Pediatrics	820	1.03%
Others	18,240	22.98%
Hospital type		
Medical center	53,680	67.62%
Regional Hospital	25,640	32.30%
District hospital	60	0.08%
Ownership		
Public hospital	20,220	25.47%
Non-profit hospital	57,760	72.77%
Private hospital	1400	1.76%

**Fig. 2:**
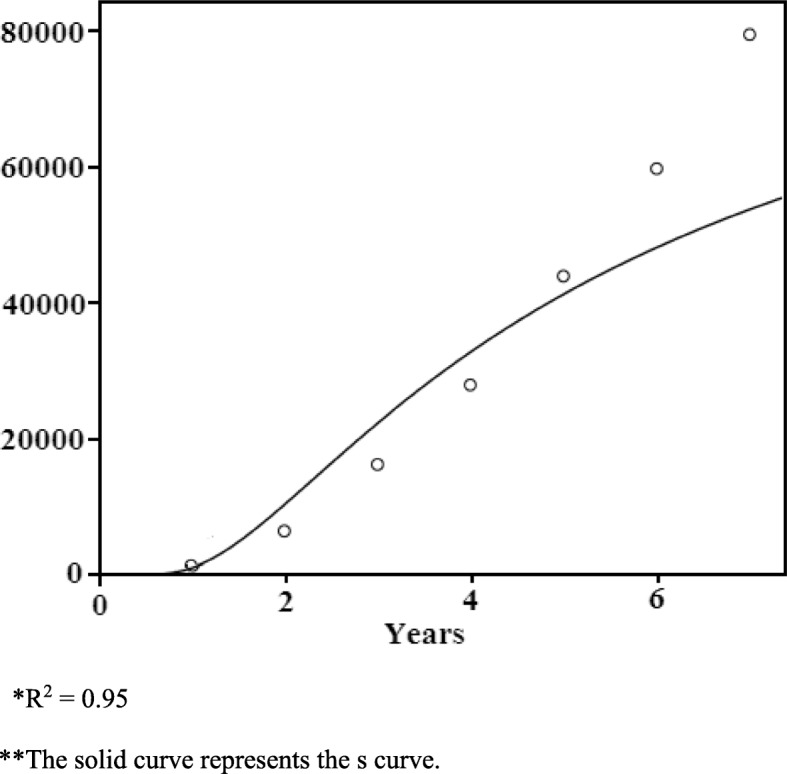
The diffusion curve for PET in terms of cumulative service volume. *R2 = 0.95. **The solid curve represents the s curve

We then examine whether the volume curve of cumulative PET services resembles an S diffusion curve. The R^2^ is 0.95 as demonstrated in Fig. 2, which means that it can fit into an S curve model; as such, the growth of cumulative PET service volume does correspond with the innovation diffusion model.

**Fig. 3:**
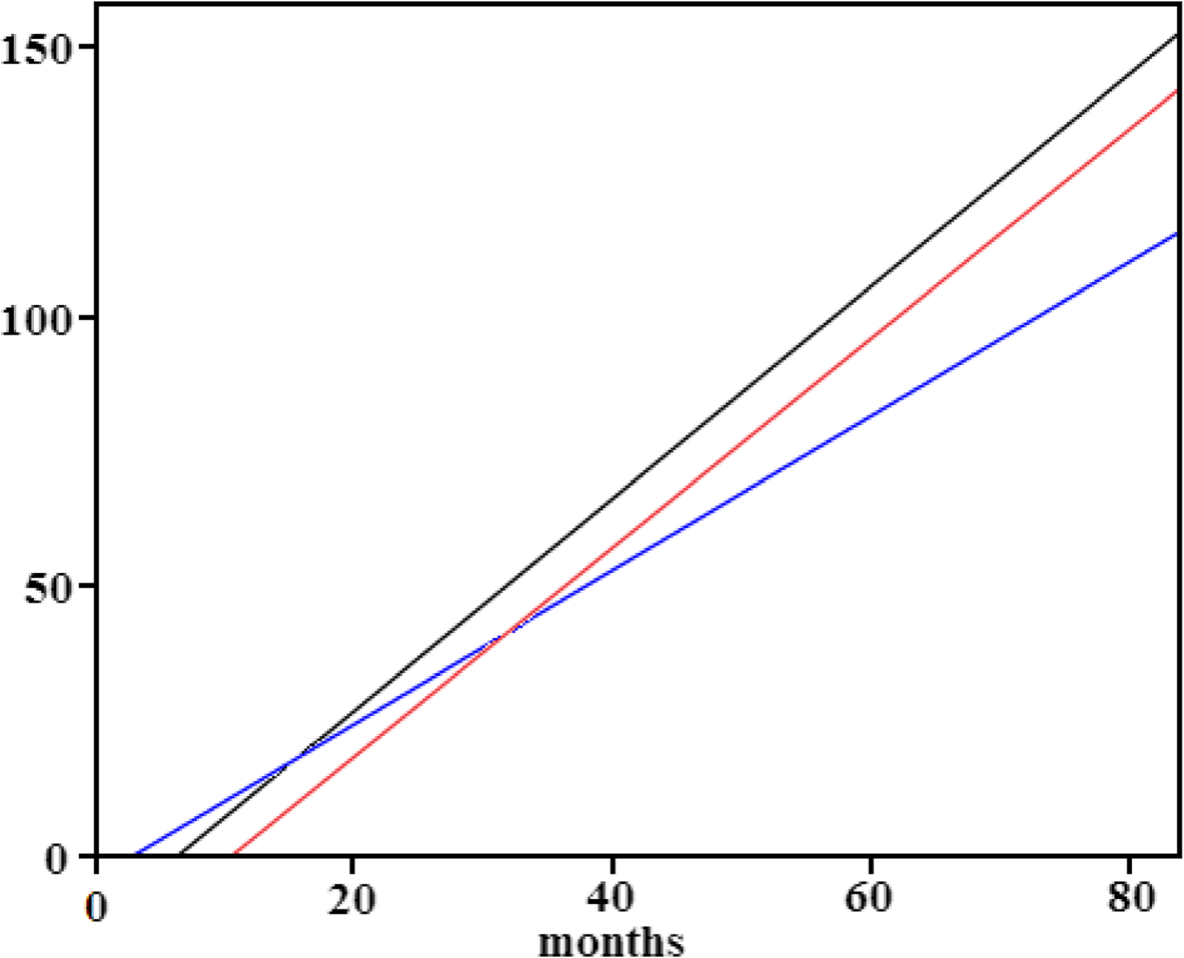
The Chou tests for the comparison of the diffusion curves of CT, MRI and PET. *X axis: months after been reimbursed by the NHI; Y axis: cumulative service volume divided by the volume of the first month. **Blue line (intercept=-4.015, slope=1.425) : The diffusion curve of CT; Black line (intercept=-12.318, slope=1.962): The diffusion curve of MRI; Red line (intercept=-20.294, slope=1.935): The diffusion curve of PET. ***Results of Chow test: between CT and MRI: *P*=0.59; between MRI and PET: *P*=0.39;between CT and PET: *P*=0.76

We further applied the cumulative service volumes of CT and MRI of from 1997 to 2003 respectively to examine by Chow’s test those of PET from 2004 to 2010. As indicated in Fig. 3, the three cumulative utilization lines demonstrate good correlation with no significant difference in their slopes which represent their growth rates. Therefore, we can infer that even though PET was reimbursed after the implementation of global budgeting, its diffusion was not deterred by this cost containment measure when compared with CT and MRI in the same time span after the inauguration of the NHI.

## Discussion

Due to the high cost of PET scans, when it was first introduced into Taiwan, it was paid primarily out of pocket by the patients or for health checkup. Therefore, the volume reimbursed by the NHI does not represent the total volume of PET services performed in Taiwan. As such our Ministry of Health and Welfare (MOHW) also conducted annual survey among hospitals to ascertain the utilization pattern of high tech services. By comparing the NHI claims with the MOHW’s annual surveys, we can see the NHI reimbursed percentage has risen from the 34.62% of 2005 to the 64.31% of 2010. More and more PET scans are provided under the NHI scheme rather than out of patients’ own pocket. But all in all, about one third of PET services are still paid out of pocket.

The price tag for PET in the NHI reimbursement schedule has been fixed at $1200 USD per examination since 2004. This price has also mostly been the market price for out of pocket patients. Taiwan’s GDP per capita has grown from $15,388 USD in 2004 to $22,540 USD in 2016 [14]. So even in 2016, paying a PET out of pocket constitutes 5.32% of an average person’s annual income. It is still a huge burden for patient have to pay out of pocket.

The service intensity amounts to 1.32 exams per thousand persons in 2010 including both provided by the NHI and not provided by the NHI. Up to 64.31% was provided by the NHI in 2010. The majority of the NHI reimbursed services were provided by the non-profit sector. Non-profit hospitals form a major sector in our hospital population and only 25.47% was prescribed by public hospitals. The findings correspond with the distribution of market shares among the variety of hospital ownership. Public hospitals only account for around 25–30% of the services in Taiwan. Most of the health care services are provided by non-public hospitals. Of the non-public hospitals, non-profit hospitals tend to be larger in terms of bed numbers and more equipped in Taiwan. That explains why non-profit hospitals have provided the majority of PET services.

From the findings of our study, we can see that global budgeting does not deter the diffusion of high tech services, such as PET, in Taiwan once it is included in the NHI reimbursement scheme with reference to the previously reimbursable scans such as MRI and CT. This finding is contrary to the prevalent arguments in the literature that prospective payment is likely to deter the diffusion of high tech and new services [3]. However, similar results have been found in a study conducted in Maryland of the United States. Their results indicate that global budgets in rural Maryland hospitals did not reduce hospital use or price-standardized spending for Medicare beneficiaries [15].

The possible explanation is that there was still fierce competition among hospitals in Taiwan under global budgeting. Although the annual budget is capped for the whole country, hospitals still need to compete for their shares in an open market with very few restrictions on patients’ choices of their health care providers. In other words, hospitals still need to purchase state of the art equipment to showcase their capabilities in order to attract patients. As such, once PET was reimbursed by the NHI, there is no stopping it.

However, the NHI did not reimburse PET service until 2004 and global budgeting has already been implemented in 2000. Under the scheme of global budgeting, the collective psyche of healthcare providers is resisting the introduction of new reimbursable items for the reason that more reimbursement items will increase the numerator and dilute the pool of money. From this perspective, we can say that the adoption of this new technology by Taiwan as a whole had been delayed due to the impact of global budgeting.

This research studied the diffusion trend of PET in the country as a whole in comparison with CT and MRI. Although the influence of some potential confounders limited by the availability of the data sets, such as percentage of out of pocket services, inpatient or outpatient, specialty etc., has been touched upon in the descriptive analyses, more studies based on the providers’ and patients’ characteristics are warranted in the future to more accurately partial out the net effect of global budgeting on the adoption of high tech services.

## Conclusion

It has always been argued that prospective payment, such as global budgeting, will deter the development of high-tech services in the healthcare industry. However, our study has found out that in the instance of PET global budgeting did not significantly change its diffusion pattern in Taiwan after it was reimbursed by the NHI in comparison with its forerunners, CT and MRI. However, our results are based on the findings after PET was adopted by the NHI. As such, global budgeting might have delayed the appearance of innovators under the NHI scheme in terms of Rogers’ diffusion of innovations theory. In conclusion, global budgeting might have made it hard for PET to get in, and yet once in it took off along the trajectory similar to all the other innovations adopted before the implementation of global budgeting.

## Abbreviations

CT: Computed Tomography
GDP: Gross Domestic Product
MOHW: Ministry of Health and Welfare
MRI: Magnetic Resonance Imaging
NHI: National Health Insurance
PET: Positron Emission Tomography
USD: United States Dollar
